# Differences in the Offensive and Defensive Actions of the Goalkeepers at Women’s FIFA World Cup 2011

**DOI:** 10.3389/fpsyg.2019.00223

**Published:** 2019-02-11

**Authors:** Pilar Sainz de Baranda, Laura Adán, Antonio García-Angulo, Maite Gómez-López, Brittany Nikolic, Enrique Ortega-Toro

**Affiliations:** ^1^Department of Physical Activity and Sport, Faculty of Sport Science, Regional Campus of International Excellence “Campus Mare Nostrum”, University of Murcia, Murcia, Spain; ^2^Department of Social Sciences Applied to Sport, Physical Activity and Leisure, Faculty of Sciences for Physical Activity and Sport (INEF), Polytechnic University of Madrid, Madrid, Spain; ^3^Department of Health, Exercise Science and Sport Management, University of Wisconsin-Parkside, Kenosha, WI, United States

**Keywords:** women’s football, match statistics, notational analysis, performance indicators, soccer

## Abstract

The aim of this study was to analyze the technical and tactical offensive and defensive actions of the goalkeepers and to determine the relationship between these actions and the qualifying results of their respective teams. The sample studied is made up of the goalkeepers (*n* = 20) of the senior national teams that participated in the FIFA Women’s World Cup in Germany 2011. A descriptive analysis was developed comparing the offensive and defensive actions in competition carried out by the goalkeepers on qualified teams (pass the group stage) with the goalkeepers on unclassified teams (not pass the group stage). For the inter-group comparison, the value of the coefficient of variation was incorporated and the effect size calculated. All data were treated with a statistical significance level of *p* < 0.05. The results show that the goalkeepers on qualified teams have higher offensive registers, as well as a higher number of passes successfully completed in different areas of the field. The goalkeepers on unclassified teams show higher defensive records such as saves inside the area, foot stops and wrong clearances among others.

## Introduction

The number of scientific investigations on women’s football specific to the topics of player characteristics and demands of the game has considerably increased in recent years due to the increased popularity of the women’s game worldwide, although they are not yet as numerous as in the case of men’s football (Martínez-Lagunas et al., 2014). However, most of the published studies have been focused on the physiological and physical attributes of female footballers, which appear to condition the way the team plays and performs during games and competition (Gabbett and Mulvey, 2008; Mohr et al., 2008).

The analysis of competition is currently a key process for improving the performance of football teams in matches and training (Carling et al., 2005; Sarmento et al., 2014). This analysis of the competition pretends to identify the strengths of the own or rival team and to have information more adequate to the complexity of collective sports (Carling et al., 2008; Agras et al., 2016). To this end, a variety of performance indicators are proposed, which are a combination of variables that help to achieve sporting success (Hughes and Bartlett, 2002; Mackenzie and Cushion, 2013). These indicators constitute an ideal profile that can be used to predict future behavior in a sporting activity (O’Donoghue, 2005). The comparative analysis over time of the performance profiles of the winning teams reveals how the styles of play evolve and identifies those variables (such as possession of the ball or blank shots) that are considered the most important in today’s football (Castellano et al., 2012; Sarmento et al., 2014).

In football, the characteristics of the players, the tactics, the rhythm of the game and play at home or abroad are the most important factors in the performance (Yamanaka et al., 1993; Bangsbo et al., 2006; Taylor et al., 2010). Hughes and Franks (2005) compared the performance of successful and unsuccessful teams at the 1990 FIFA World Cup, finding greater possession and more shots on goal from successful teams. Several studies have found differences in the individual and collective technical and tactical patterns of the teams with the highest sports performance (Yang et al., 2018). In this line, Folgado et al. (2015) point to the level of tactical development, the pace of play and player fatigue as determining factors in performance. In this way, the specific position of the player affects the technical, tactical and physiological performance of the players in competition (Reilly, 1997; Di Salvo et al., 2008). Studies have found large differences in the physiological efforts and play actions of different specific positions in football (Rienzi et al., 2000; Di Salvo et al., 2008).

A few studies have focused on match analysis of women’s football. Soroka and Bergier (2010) studied the characteristics of attack and defense actions to win or lose in women’s football. Hewitt et al. (2014) reported a different game activity between higher and lower ranked teams in women’s football. Pollard and Gómez (2014) analyzed the advantage of playing at home in the European women’s leagues, comparing them to the men’s leagues, concluding that the advantage is greater in the men’s leagues. On the other hand, Ibañez et al. (2018) reported a different game activity between higher and lower ranked teams in women’s football. However, the studies have not specifically analyzed the characteristics of the goalkeeper’s actions and their impact on the team performance.

The goalkeeper is the most specialized position in football. The goalkeeper’s primary role in soccer is to protect his/her team’s goal, whilst a secondary purpose lies in ball distribution during the initiation of an attack. As the objective of football is to out-score the opposition, it stands to reason that the demands placed upon goalkeepers have the potential to directly influence the outcome of a match (Seaton and Campos, 2011). Indeed, as the only players permitted to legally handle the ball (when inside the penalty area) whilst the game is “live,” their positional role is not akin to that of other outfield playing positions (Van Der Kamp, 2006; Di Salvo et al., 2008; Fariña et al., 2013; Almeida et al., 2016).

White et al. (2018) made a review of current literature about match-play and performance test responses of football goalkeepers. This review summarized the available literature pertaining to the performance responses of football goalkeepers and concluded that football goalkeepers demonstrate different physiological profiles from outfield players (i.e., superior jump performance, reduce VO2max values, slower sprint times). Similarly, there are differences in physiological, anthropometric and technical parameters between high-performance and amateur goalkeepers (Rebelo et al., 2013).

In relation with the technical and tactical analysis of the goalkeeper’s actions some studies have used observational techniques to identify the type of activities performed by goalkeepers during match play. In youth players Sainz de Baranda et al. (2005c), analyzed the goalkeeper’s defensive actions in the 7-a-side football format. At this same age, Ortega-Toro et al. (2018) found differences in the goalkeeper’s offensive and defensive actions between the formats of Fútbol-5 and Fútbol-8. In high-performance football, Sainz de Baranda et al. (2008), during the 2002 World Cup, reported that international goalkeepers performed 23 defensive technical actions over 90 min, of which the most frequent actions were saves. Other studies of Spanish professional goalkeepers reported a lower incidence of saves, however, these studies reinforce the defensive role of football goalkeepers (Liu et al., 2015). Liu et al. (2015) examined the performance of goalkeepers by considering three situational variables (opposition, result, and location). Along the same lines, Villemain and Hauw (2014) identified the game actions that generate the most uncertainty for goalkeepers.

Peráček et al. (2017), during the European Championship U17, found that demands on the goalkeeper’s game performance are high. However, they found out that at present there has been a change in the ratio between defensive and offensive game activities in favor of offensive.

Then, goalkeepers need to participate/contribute effectively to the implementation of the team’s game model and not only stay on goalpost to defend/stop the shots. Goalkeepers need to be able to play with their feet, actively participating in the offensive phase, mainly in the distribution of the ball (participates in build up play and involved as much as possible in shaping the play when team is in possession). Therefore, Sainz de Baranda et al. (2005b) point out the importance of analyzing the participation of the goalkeeper both in the defensive phase and in the offensive phase.

The aim of the current study was to analyze technical and tactical offensive and defensive performance of goalkeepers from the national teams participating in the 2011 FIFA Women’s World Cup in Germany, comparing the actions of the goalkeepers on qualified teams (pass the group stage) with the goalkeepers on unclassified teams (do not pass the group stage).

## Materials and Methods

The sample included 20 goalkeepers from the 16 national teams that participated in 32 matches of the 2011 FIFA Women’s World Cup in Germany (age: 28.9 ± 4.2 years; height: 172.5+6.5 cm; weight: 62.3 ± 6.3 kg; years of experience: 11.23 ± 4.23 years). Teams were divided in two groups in relation to advanced (classified teams) or not advanced (unclassified teams) from the group stage of the tournament. The first round, or group stage, sees the sixteen teams divided into four groups of four teams. The knockout stage comprises the eight teams that advanced from the group stage of the tournament. The teams who passed the first round were: Germany, Japan, Sweden, Australia, England, France, Brazil and United States (these teams were the classified teams). The unclassified teams were: Equatorial Guinea, Nigeria, Korea DPR, Norway, Canada, Mexico, New Zealand, and Colombia.

Statistics used in the study were made available by OPTA Sportsdata Spain Company (Madrid). The reliability of tracking system (OPTA Client System) has been verified by Liu et al. (2013) which showed a high level of inter-operator reliability using the system to track goalkeeper’s match actions (weighted kappa for two tested goalkeepers: 0.86 and 0.92). The Company maintained the anonymity of players and teams following European Data Protection Law. The study was approved by the Ethics Committee of University of Murcia (ID 1944/2018).

**FIGURE 1:**
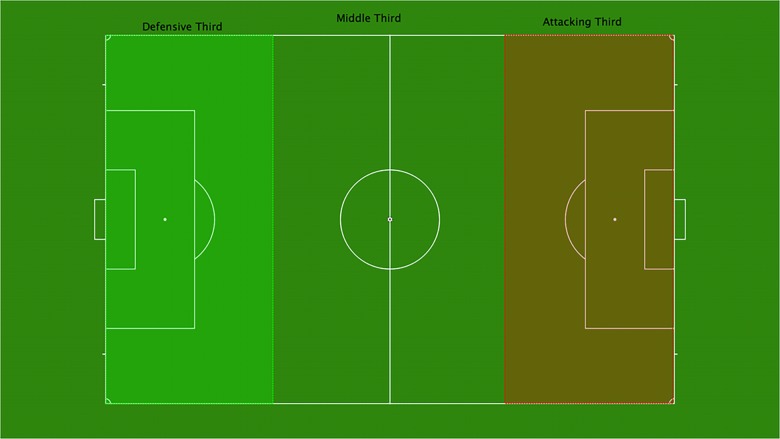
Field zones used for establishing the pass length in the offensive actions of the goalkeeper.

Based on the review and analysis of available literature in the performance analysis of football, the following match performance indicators were chosen for analyses the goalkeepers’ intervention. The variables were divided into two main categories (offensive and defensive actions) (see Figure 1). The offensive actions were divided in pass zones and types of passes (see Table 1) and the defensive actions were divided in goals and shots received, types of saves and basic goalkeeper’s actions (see Table 2).

**Table 1:** List of goalkeeper offensive actions and definitions.

Variables	Technical/tactical action	Definition
Pass zones	Attempted passes in own half (APOH)	Any intentional played ball from one player to another in own half of field
	Successful passes in own half (SPOH)	Any intentional played ball from one player to another in own half of field
	Attempted passes in opponent’s half (APOP)	Any intentional played ball from one player to another in the attacking half of the pitch (opposite field)
	Successful passes in opponent’s half (SPOP)	Accurate passes in the attacking half of the pitch (opposite field)
	Attempted passes in defensive third field (APDT)	Any intentional played ball from one player to another in defensive third field
	Successful passes in defensive third field (SPDT)	Accurate passes in defensive third field
	Attempted passes in middle third field (APMT)	Any intentional played ball from one player to another in middle third field
	Successful passes in middle third field (SPMT)	Accurate passes in middle third field
	Attempted passes in offensive third field (APOT)	Any intentional played ball from one player to another in offensive third field
	Successful passes in offensive third field (SPOT)	Accurate passes in offensive third field
Types of passes	Short passes (SP)	Passes over a distance less than 35 yards or 32 meters
	Success in short passes (SSP)	Accurate passes over a distance less than 35 yards or 32 meters
	Long passes (LP)	Accurate passes that have a distance greater than 35 yards or 32 meters
	Success in long passes (SLP)	Accurate passes that have a distance greater than 35 yards or 32 meters
	Passes received (PR)	Sending and receiving the ball between two players of the same team
	Passes to the opponent (POP)	Passes to the opponent
	Team’s own forward passes (TOFP)	Team’s own forward passes
	Team’s own diagonal passes (TODP)	Team’s own diagonal passes
	Passes forward to the opponent (PFO)	Passes forward to the opponent
	Passes with a fast moving ball (PMB)	Style of play in which the ball is passed on or distributed after only one or two touches
	Successful passing with a fast moving ball (SPMB)	Accurate Passes with a fast moving ball
	Long ball goal kick (LBGK)	Kick that is used to return into play from inside the goal area (greater than 35 yards or 32 meters); awarded to the defending team when a ball that crossed the goal line was last touched by a player on the attacking team.
	Successful long ball goal kick (SLBGK)	Accurate long ball goal kick (greater than 35 yards or 32 meters)
	Short goal kicks (SGK)	Kick that is used to return into play from inside the goal area (less than 35 yards or 32 meters); awarded to the defending team when a ball that crossed the goal line was last touched by a player on the attacking team.
	Successful short goal kick (SSGK)	Accurate short goal kick (less than 35 yards or 32 meters)

**Table 2:** List of goalkeeper defensive actions and definitions.

Variables	Technical/tactical action	Definition
Goals and shots received	Goals allowed (GA)	Total goals scored by the opposition
	Goals allowed inside the area (GAIA)	Total goals conceded from a shot inside the area
	Goals allowed outside the area (GAOA)	Total goals conceded from a shot outside the area
	Shots allowed (SA)	Total shots made by the opposition
	Shots allowed inside the area (SAIA)	Total shots made inside the area
	Shots allowed outside the area (SAOA)	Total shots made outside the area
	Goals allowed in fast play (GAFP)	Total goals scored by the opposition in fast play. Counter attack.
	Goals allowed in clear play (GACP)	Total goals scored by the opposition in clear play. Open play attack.
	No goals are conceded (NG)	No goals conceded to the rival team
Types of savesSaves: A goalkeeper preventing the ball from entering the goal with any part of his body when facing an intentional attempt from an opposition player	Saves inside the area (SIA)	Saves inside de area
	Saves outside the area (SOA)	Saves outside de area
	Set-piece saves (SPS)	Goalkeeper saves a shot from set-piece (corner kick, free kick, or throw-in)
	Air saves (AS)	Total goalkeeper air saves
	Standing saves (SS)	Goalkeeper saves a shot by standing and deflecting/parrying to safety saves away from starting position
	Saves on site (SOS)	Total goalkeeper saves on site
	Fingertip saves (FS)	Goalkeeper save using his/her fingertips
	Palm hand saves (PHS)	Goalkeeper save using his/her palm hand
	Foot saves (FOS)	Goalkeeper save using his/her feet
	Body saves (BS)	Goalkeeper save using his/her body
	Oriented clear (OC)	Attempt made by the goalkeeper to get the ball out of the danger zone
	Badly oriented clear (BOC)	Clearing the ball from danger by kicking it up field or out of bounds. The kick has no intended receiver and is usually done to relieve pressure in the goal area.
	Block saves (BLOS)	Block a shot that would have resulted in a goal
	Two-step saves (2STS)	Ball secured but not on first attempt
Basic Goalkeeper’s actions	Total clearance (TC)	Total number of times the ball is clearances defensively. The goalkeeper clears the ball in difficult situations when he is not sure he can catch it
	Blocks (BLK)	A goalkeeper blocks the ball from reaching its target
	Punches (PU)	A high ball that is punched clear by the goalkeeper. The keeper must have a clenched fist and attempting to clear the high ball rather than claim it.
	Drops (DRP)	A high ball where the goalkeeper tries to catch the ball, she gets her hands on the ball but drops it from grasp.
	Hand-blocks balls (HBB)	A goalkeeper blocks a shot with her hand.
	Recoveries (RE)	When the goalkeeper takes possession of a loose ball and successfully keeps possession for at least two passes or an attacking play

### Statistical Analysis

First, a descriptive analysis based on goalkeeper actions (mean and standard deviation) was performed. In addition, the value of the coefficient of variation (CV) for each of the variables was incorporated in order to know the variability or stability of each action of the goalkeeper. After that, the mean difference test (T student for independent samples) was carried out, analyzing the variables and using as grouping variables the classified teams (teams that had passed the first round; n = 8) against the non-classified teams (teams that had not passed the first round; n = 8). To know the magnitude of the differences found, the size of the effect (ES) was calculated using the Cohen’s d (1988) considering the values as small effect (d < 0.2), medium effect (0.2 ≤ d < 0.6), high effect (0.6 ≤ d < 1.2) and strong effect (d > 1.2). Statistical analyses were performed using the IBM SPSS Statistics 21.0 statistical package (IBM Corp., Armonk, NY, United States) and the statistical significance was established at p < 0.05.

## Results

Table 3, shows the differences between classified and unclassified teams, and total values of the variables of the pass areas.

**Table 3:** Offensive actions of the goalkeepers.

	Unclassified			Classified			Total			*P*	ES	E^∗∗^	CI (90%)	
	Mean	*SD*	CV	Mean	*SD*	CV	Mean	*SD*	CV				Lw	Upp
APOH	13.23	6.04	45.60	14.78	4.65	31.46	14.01	5.27	37.61	0.57	0.27	M	−0.54	1.11
SPOH	6.33	3.87	61.13	9.53	2.95	30.95	7.93	3.71	46.78	0.08	0.88	H	0.04	1.79
APOP	10.78	6.28	58.25	13.26	6.31	47.58	12.02	6.21	51.66	0.44	0.37	M	−0.44	1.22
SPOP	2.86	1.90	66.43	4.72	2.48	52.54	3.79	2.34	61.74	0.11	0.80	H	−0.03	1.69
APDT	8.55	4.46	52.16	8.91	2.62	29.40	8.73	3.53	40.43	0.84	0.09	S	−0.73	0.92
SPDT	8.03	4.23	52.67	8.38	2.47	29.47	8.20	3.35	40.85	0.84	0.10	S	−0.72	0.92
APMT	11.81	5.25	44.45	14.70	4.80	32.65	13.26	5.08	38.31	0.27	0.54	M	−0.28	1.41
SPMT	4.53	2.16	47.68	6.55	1.95	29.77	5.54	2.24	40.43	0.06	0.93	H	0.09	1.84
APOT	3.65	2.19	60	4.44	3.21	72.29	4.04	2.69	66.58	0.57	0.27	M	−0.54	1.11
SPOT	0.35	0.31	88.57	1.25	1.11	88.80	0.80	0.91	113.75	0.05	1.04	H	0.20	1.98

*Differences between classified and unclassified, and total values of the pass areas. ^∗^Differences statistically significant. APOH, attempted passes in own half; SPOH, successful passes in own half; APOP, attempted passes in opponent’s half; SPOP, successful passes in opponent’s half; APDT, attempted passes in defensive third field; SPDT, successful passes in defensive third field; APMT, attempted passes in middle third field; SPMT, successful passes in middle third field; APOT, attempted passes in offensive third field; SPOT, successful passes in offensive third field. ^∗∗^ E = effect [small effect (d < 0.2) = S; medium effect (0.2 ≤d < 0.6) = M; high effect (0.6 ≤d < 1.2) = H; strong effect (d > 1.2) = ST].*

The results in Table 3 show that the mean number of passes attempted made by the goalkeeper and their success is always higher in the qualified teams (teams that have passed the group stage). Conversely, the coefficient of variation, for the majority of unclassified teams is higher, except for the variables: attempted passes in the offensive third and the successful passes in the offensive third field. It is also noteworthy that none of the variables show any significant differences between classified and unclassified teams; There exists a trend toward statistical significance in the successful passes in the middle third field (p = 0.06; ES = 0.93; high effect) and in the successful passes in the offensive third field (p = 0.05; ES = 1.04; high effect).

The results shown in Table 4 show the type of passes that goalkeepers make. In all the variables the mean is higher in the classified teams. In addition, the actions that are most performed by both the classified and unclassified teams are long passes with a mean of 19.38+6.40 for classified and 15.85+6.79 for unclassified teams and passes on the contrary with a mean of 13.30+6.36 for classified and 10.78+6.28 for unclassified teams. The coefficient of variation is higher in most unclassified teams. Finally, it is observed that there are statistically significant differences in passes with a fast moving ball (p = 0.027; ES = 1.16; high effect) and in the successful passing with a fast moving ball (p = 0.028; ES = 1.16; high effect).

**Table 4:** Offensive actions of the goalkeepers.

	Unclassified			Classified			Total			*P*	ES	E^∗∗^	CI (90%)	
	Mean	*SD*	CV	Mean	*SD*	CV	Mean	*SD*	CV				Lw	Upp
SP	8.18	4.05	49.51	8.67	3.03	34.94	8.42	3.46	41.09	0.78	0.13	S	−0.69	0.96
SSP	7.24	3.77	52.07	7.77	2.55	32.81	7.51	3.12	41.54	0.74	0.16	S	−0.66	0.99
LP	15.83	6.79	42.89	19.38	6.40	33.02	17.60	6.63	37.67	0.30	0.51	M	−0.31	1.37
SLP	5.68	2.59	45.59	8.41	2.24	26.63	7.04	2.73	38.77	0.40	1.07	H	0.22	2.00
PR	4.47	3.70	82.77	8.49	4.15	48.88	6.48	4.33	66.82	0.06	0.97	H	0.13	1.89
POP	10.78	6.28	58.25	13.30	6.36	47.81	12.04	6.24	51.82	0.43	0.38	M	−0.44	1.22
TOFP	8.94	3.65	40.82	9.11	4.15	45.55	9.03	3.78	41.86	0.92	0.04	S	−0.78	0.87
TODP	9.01	5.57	61.82	9.94	2.58	25.95	9.47	4.22	44.56	0.67	0.20	M	−0.61	1.04
PFO	10.78	6.28	58.25	13.30	6.36	47.81	12.04	6.24	51.82	0.43	0.39	M	−0.44	1.22
PMB	6.85	4.72	68.90	12.77	4.90	38.37	9.81	5.56	56.67	0.02^∗^	1.16	H	0.31	2.12
SPMB	4.83	3.36	69.56	8.59	2.77	32.24	6.71	3.55	52.90	0.02^∗^	1.16	H	0.30	2.11
LBGK	7.15	3.35	46.85	8.81	2.32	26.33	7.98	2.92	36.59	0.26	0.54	M	−0.28	1.41
SLBGK	3.50	2.05	58.57	4.49	0.69	15.36	3.99	1.57	39.34	0.23	0.61	H	−0.21	1.48
SGK	8.19	3.61	44.07	5.29	2.21	41.77	6.74	3.26	48.36	0.07	0.92	H	0.08	1.83
SSGK	7.51	3.51	46.73	4.95	2.12	42.82	6.23	3.10	49.75	0.09	0.83	H	0.002	1.73

*Differences between classified and unclassified, and total values of pass categories. ^∗^Differences statistically significant. SP, short passes; SSP, success in short passes; LP, long passes; SLP, success in long passes; PR, passes received; POP, passes to the opponent; TOFP, team’s own forward passes; TODP, team’s own diagonal passes; PFO, passes forward to the opponent; PMB, passes with a fast moving ball; SPMB, successful passing with a fast moving ball; LBGK, long ball goal kick; SLBGK, successful long ball goal kick; SGK, short goal kicks; SSGK, successful short goal kick. ^∗∗^ E = effect [small effect (d < 0.2) = S; medium effect (0.2 ≤d < 0.6) = M; high effect (0.6 ≤d < 1.2) = H; strong effect (d > 1.2) = ST].*

The results in Table 5 show the goals and shots conceded by the goalkeepers. In almost all variables, the mean number of goals and shots allowed is higher in unclassified teams, especially in shots allowed (SA – 5.51 ± 0.97) and shots allowed inside the area (SAIA – 3.57 ± 0.89). The exceptions lie in goals from outside the area (GAOA) (0.21 ± 0.31 vs. 0.24 ± 0.20) and the number of times they do not concede a goal (NG) (0.16 ± 0.17 vs. 0.32 ± 0.21) which is higher in classified teams. In addition, there are statistically significant differences in the variable shots allowed (p = 0.001; ES = 1.92; strong effect) and in the variable shots allowed inside the area (p = 0.001; ES = 1.86; strong effect).

**Table 5:** Defensive actions of the goalkeepers.

	Unclassified			Classified			Total			*P*	ES	E^∗∗^	CI (90%)	
	Mean	*SD*	CV	Mean	*SD*	CV	Mean	*SD*	CV				Lw	Upp
GA	1.66	0.67	40.36	1.10	0.42	38.18	1.38	0.61	44.20	0.07	0.95	H	0.11	1.86
GAIA	1.45	0.69	47.58	0.86	0.51	59.30	1.16	0.66	56.89	0.07	0.92	H	0.08	1.83
GAOA	0.21	0.31	147.61	0.24	0.20	83.33	0.22	0.25	113.63	0.81	0.11	S	−0.71	0.94
SA	5.51	0.97	17.60	3.53	0.98	27.76	4.52	1.39	30.75	0.001ˆ*	1.92	ST	0.97	3.04
SAIA	3.57	0.89	24.92	2.00	0.69	34.50	2.79	1.12	40.14	0.001ˆ*	1.86	ST	0.92	2.97
SAOA	1.94	0.63	32.47	1.53	0.70	45.75	1.73	0.68	39.30	0.242	0.58	M	−0.24	1.45
GAFP	0.19	0.21	110.52	0.07	0.14	200.00	0.13	0.18	138.46	0.21	0.64	H	−0.19	1.51
GACP	1.08	0.53	49.07	0.67	0.51	76.11	0.88	0.55	62.50	0.13	0.75	H	−0.08	1.63
NG	0.16	0.17	106.25	0.32	0.21	65.62	0.24	0.20	83.3	0.99	0.79	H	−0.04	1.69

*Differences between classified and unclassified with respect to goals and shots received. ^∗^Differences statistically significant. GA, goals allowed; GAIA, goals allowed inside the area; GAOA, goals allowed outside the area; SA, shots allowed; SAIA, shots allowed inside the area; SAOA, shots allowed outside the area; GAFP, goals allowed in fast play; GACP, goals allowed in clear play; NG, no goals conceded. ^∗∗^ E = effect [small effect (d < 0.2) = S; medium effect (0.2 ≤d < 0.6) = M; high effect (0.6 ≤d < 1.2) = H; strong effect (d > 1.2) = ST].*

The results included in Table 6 show the types of saves made by the goalkeepers. In almost all the variables the mean number of actions is higher in the goalkeepers of the unclassified teams, with the exception of the variables set-piece saves (SPS) (0.08 ± 0.15 vs. 0.10 ± 0.18) and body saves (BS) (0.10 ± 0.20 vs. 0.14 ± 0.20), which are higher in classified teams. The CV is higher in almost all classified teams. In addition, statistically significant differences are observed in more than one variable: saves inside the area (SIA) (p = 0.010; ES = 1.41; strong effect), standing saves (SS) (p = 0.009; SE = 1.42; strong effect); palm hand saves (PHS) (p = 0.009; ES = 1.43; strong effect); and badly oriented clears (p = 0.010; ES = 1.39; strong effect).

**Table 6:** Defensive actions of the goalkeepers.

	Unclassified			Classified			Total			*P*	ES	E^∗∗^	CI (90%)	
	Mean	*SD*	CV	Mean	*SD*	CV	Mean	SD	CV				Lw	Upp
SIA	2.08	0.73	35.09	1.08	0.61	56.48	1.58	0.83	52.53	0.01ˆ*	1.41	ST	0.52	2.41
SOA	1.73	0.70	40.46	1.29	0.61	47.28	1.51	0.68	45.03	0.20	0.63	H	−0.19	1.51
SPS	0.08	0.15	187.5	0.10	0.18	180.00	0.09	0.16	177.77	0.80	0.11	S	−0.70	0.94
AS	0.90	0.32	35.55	0.52	0.38	73.07	0.71	0.39	54.92	0.05	1.02	H	0.18	1.95
SS	1.95	0.32	16.41	1.18	0.65	55.08	1.56	0.63	40.38	0.009ˆ*	1.42	ST	0.54	2.42
SASP	0.28	0.38	135.71	0.17	0.15	88.23	0.22	0.28	127.27	0.43	0.36	M	−0.46	1.20
SOS	0.69	0.73	105.79	0.51	0.36	70.58	0.60	0.56	93.33	0.54	0.30	M	−0.52	1.14
FS	0.17	0.36	211.76	0.03	0.09	300.00	0.10	0.26	260.00	0.32	0.50	M	−0.31	1.36
PHS	3.58	1.04	29.05	2.18	0.79	36.23	2.88	1.15	39.93	0.009ˆ*	1.43	ST	0.55	2.44
FOS	0.13	0.17	130.76	0.04	0.08	200.00	0.08	0.14	175.00	0.24	0.64	H	−0.18	1.51
BS	0.10	0.20	200	0.14	0.20	142.85	0.12	0.19	158.33	0.75	0.19	S	−0.63	1.02
OC	0.95	0.65	68.42	0.66	0.57	86.36	0.80	0.61	76.25	0.35	0.45	M	−0.37	1.30
BOC	0.86	0.37	43.02	0.39	0.26	66.66	0.63	0.40	63.79	0.01ˆ*	1.39	ST	0.51	2.39
BLOS	0.90	0.65	72.22	0.71	0.46	64.78	0.80	0.55	68.75	0.50	0.32	M	−0.50	1.16
2STS	0.94	0.84	89.36	0.57	0.64	112.28	0.76	0.75	98.68	0.34	0.47	M	−0.35	1.32

*Differences between classified and unclassified and types of saves. ^∗^Differences statistically significant. SIA, saves inside the area; SOA, saves outside the area; SPS, set-piece saves; AS, air saves; SS, standing saves; SASP, saves away from starting position; SOS, saves on site; FS, fingertip saves; PHS, palm hand saves; FOS, foot saves; BS, body saves; OC, oriented clear; BOC, badly oriented clear; BLOS, block saves; 2STS, two-step saves. ^∗∗^ E = effect [small effect (d < 0.2) = S; medium effect (0.2 ≤d < 0.6) = M; high effect (0.6 ≤d < 1.2) = H; strong effect (d > 1.2) = ST].*

Table 7 shows the basic defensive goalkeeper’s actions. In all actions the mean obtained in the variables is higher in the unclassified teams with the exception of the variable of recoveries (RE) (11.38 ± 1.30 vs. 12.17 ± 3.35) that is higher in the teams classified. It is also observed that the coefficient of variation (CV) is higher in classified teams, except for the variable total clearance (TC) (60.75% for unclassified teams) and punches (PU) (86.71% for unclassified teams). The variable recoveries is the most performed variable by both classified and unclassified goalkeepers, followed by the action of hand-blocks balls (HBB). In addition, there are statistically significant differences in the variable drops (DRP) (p = 0.006; ES = 1.49; strong effect).

**Table 7:** Defensive actions of the goalkeepers.

	Unclassified			Classified			Total			*P*	ES	E^∗∗^	CI (90%)	
	Mean	*SD*	CV	Mean	*SD*	CV	Mean	*SD*	CV				Lw	Upp
TC	1.86	1.13	60.75	1.43	0.83	58.04	1.65	0.98	59.39	0.39	0.41	M	−0.41	1.26
BLK	1.35	0.82	60.74	1.17	0.74	63.24	1.26	0.76	60.31	0.06	0.22	M	−0.60	1.05
PU	1.43	1.24	86.71	1.14	0.78	68.42	1.28	1.01	78.90	0.58	0.26	M	−0.55	1.10
DRP	0.61	0.29	47.54	0.21	0.21	100	0.41	0.32	78.04	0.006ˆ*	1.49	ST	0.60	2.51
HBB	7.98	0.78	9.77	7.40	2.22	30.00	7.69	1.64	21.32	0.50	0.33	M	−0.49	1.17
RE	11.38	1.30	11.42	12.17	3.35	27.52	11.77	2.49	21.15	0.54	0.29	M	−0.52	1.13

*Differences in the basic goalkeeper’s actions between classified and unclassified. ^∗^Differences statistically significant. TC, total clearance; BLK, blocks; PU, punches; DRP, drops; HBB, hand-blocks balls; RE, recoveries. ^∗∗^ E = effect [small effect (d < 0.2) = S; medium effect (0.2 ≤d < 0.6) = M; high effect (0.6 ≤d < 1.2) = H; strong effect (d > 1.2) = ST].*

## Discussion

The aim of this study was to examine the technical and tactical performance of goalkeepers of the senior teams participating in the Women’s World Cup Germany 2011, differentiating between the teams who passed the group stage and the others.

The analysis of performance indicators in sports has direct practical implications. As Higham et al. (2014) note, reference values can assist in understanding variability in team performance, can aid coaches in establishing quantifiable objectives for training and performance, and can help when evaluating the efficacy of training interventions and tactical changes (Daza et al., 2017). Knowledge of performance indicators can also be used to create performance profiles to predict team behaviors and performance outcomes (Wagner et al., 2014).

One of the first works carried out with the objective of analyzing the technical-tactical performance of the football goalkeepers was that of Sainz de Baranda et al. (2008). The purpose of this study was to examine the characteristics of goalkeepers’ defense interventions in parallel with the type of opponent attack. Results related to goalkeepers’ defense showed that the penalty area was the zone most often used, and the defensive actions most frequently used were the save (9.96+3.8), followed by foot control (6.5+4.2), and the clear out (2.9+1.8). In total, Sainz de Baranda et al. (2008) observed a mean of 23.4 defensive technical actions per match. This results are similar to that reported by Sainz de Baranda and Serrato (2000) for the 1998 World Cup (22.1 actions per match) but lower than that reported by Sainz de Baranda (2002) for the 2000 European Championship (28.31 actions per match).

In the current study (FIFA, 2011), we observed a mean of 24.06 defensive technical actions per match, with 12.29 saves and 11.77 recoveries. The results of the current study and the others studies reinforce that the main defensive role of soccer goalkeepers is preventing scoring opportunities and confirm that these events occur relatively infrequently during a match, although they may be modulated by various contextual factors (Liu et al., 2015).

In the current study, results showed that there were differences in some of the match performance indicators for goalkeepers. Goalkeepers on classified teams allowed fewer goals, shots and made fewer defensive actions, saves inside the area, standing saves, palm hand saves and badly oriented clears. This is possibly due to the fact that high level teams were subjected to less attacking play from the opponents, whereas the opposite happened to goalkeepers of low level teams.

Similar findings were found by Liu et al. (2015) when league standing was used to group Spanish La Liga clubs into high-, intermediate-, and low-standard teams. The analysis of the results showed the goalkeepers on high-standard teams (i.e., top six league positions) made fewer saves than those on low-standard teams and also performed fewer touches of the ball, passes, interceptions, clearances and catches. With regard to the influence of opposition, goalkeepers on low-and intermediate-standard teams made more saves when facing a high-standard opposition than when facing other low-standard teams. Conversely, goalkeepers on high-standard teams made more saves when facing a low-standard opposition than when facing intermediate-or other high-standard teams. Such counterintuitive findings may be attributable to differences in playing style/formation when high-standard teams face a lesser opposition, whereby adopting a more expansive approach may create opportunities for the opposition to counter-attack.

It is interesting to take account that the goalkeepers on unqualified teams allowed more shots, more shots inside the area, and more goals inside the area, with statistically significant differences. Some studies show that the percentage of shots fired in a match is similar both inside and outside the area (Sainz de Baranda et al., 2008). But, with relation to the number of goals allowed, other authors claim that the most goals are scored in the area (Yagüe and Paz, 1995; Romero et al., 1997). Gómez (1999) found that 66.9% of the goals scored in the Spanish First Division in 1998–1999 were scored from inside the penalty area. In this same line, Park et al. (2016) report that 89% of goals are scored from inside the penalty area.

To develop a defensive game, the goalkeeper should coordinate his or her actions with the defensive players on the team and adapt the defensive strategy to counter the opposition. In the FIFA Women’s World Cup Germany 2011, the most successful teams were capable of moving their defensive block around 30 meters up and down the pitch without losing their balance or increasing the distances between players. Within their defensive block, they hunted for the ball and supported each other to win the ball back. Another important aspect of the successful teams (all four semi-finalists) was the fact that they had the goalkeeper playing as a libero behind the defense when the defensive block was positioned high up the pitch (FIFA, 2011). This may be one reason why the goalkeepers on classified teams made more recoveries than the goalkeepers on unclassified teams.

Another difference between the goalkeepers on classified and unclassified teams were the number of badly oriented clears and the number of drops. The higher number of oriented clears and badly oriented clears made by the goalkeepers on unclassified teams could be related with the playing style of the opponent. For example, Tenga et al. (2010) showed that the goalkeeper’s recovery is associated with counterattack. In the same way, the number of drops can be conditioned to the playing style of the opponent (crosses, corners, aerial success, etc,). The variables related to the opposing team’s attacks, related to passing and organizing, ball possession, pass, successful pass and cross were variables that had been found to discriminate winning and losing teams in UEFA Championship League (Lago-Peñas et al., 2011) and Spanish Professional Football League (Lago-Peñas and Dellal, 2010).

On the other hand, with relation to the offensive actions of the goalkeepers, it is important to know that some research has shown decreasing frequency of defensive game activities in favor of offensive game activities (Sainz de Baranda et al., 2005b). Currently, the goalkeeper not only has a defensive role, but also possesses an offensive role very important in the implementation of the team’s game model.

Sainz de Baranda et al. (2005b) examined the characteristics of goalkeepers’ offensive interventions in 56 matches of the 2002 World Cup in Korea and Japan. Sainz de Baranda et al. (2005b) observed a mean of 30.3 ± 7.9 actions per goalkeeper in a match, and the offensive actions most frequently used were the kick pass (11.31 ± 5.3) and the goal kick (9.56+2.2). In relation to length, Sainz de Baranda et al. (2008) observed a predominance of the long actions (62.19%), with an average number of 19 ± 8.9, while the short actions have an average 11.53 ± 6.84. With respect to orientation, the central zone was the most used (41.5%), followed by the right zone (29.3%) and the left zone (29.2%). When it comes to precision, 48.3% of the actions obtained direct precision, 13.9% obtained possession after rejection and 37.7% of the time ball possession was lost. Therefore, a total of 63% of the attacks started by the goalkeeper allow for continuation of ball possession.

The results of the Sainz de Baranda et al. (2005b) study indicated that the interventions in the attack have increased as the years pass. Higher data in this study (30.3 actions) than in previous studies was observed. Sainz de Baranda (2002) in the European Championship in Holland and Belgium in 2000 and Sainz de Baranda and Serrato (2000) in the World Cup in France in 1998 registered an average of 28.48 and 25.96 actions, and Yagüe and Martin (1995) in the World Cup in the United States in 1994 registered an average of 25.3 actions, respectively.

In the current study (FIFA, 2011), we observed a mean of 26.03 offensive actions per match, with 14.01 passes attempted in the own half of the field (short pass) and 12.02 passes attempted in the opposite field (long pass). Furthermore, results showed that there were differences in some of the match performance indicators for goalkeepers. Goalkeepers on classified teams attempted more long passes, more passes in the opponent’s half of field, more passes in the middle third field and more passes in the offensive third field. Overall the differences are in the success of ball distribution. The results showed that in all the variables the goalkeepers on classified teams have more precision with a high magnitude of the effect. Furthermore, there are statistically significant differences in the variables related with passes with a fast moving ball in motion. The results showed that goalkeepers on classified teams made more passes with a fast moving ball in motion than the goalkeepers on unclassified teams and there were differences between goalkeepers in terms of success too, with higher rates of successful passing with a fast moving ball.

Similar findings were found by Szwarc et al. (2010) and Seaton and Campos (2011) that suggested that there were differences between goalkeepers from different levels in terms of ball distribution and success of performance indicators.

As indicated by the FIFA in the technical report and statistics of the Women’s World Cup Germany 2011, the most-complete goalkeepers in this tournament became the team’s first point of attack after gaining possession of the ball. They were skilful in distributing the ball with their hands or their feet quickly and effectively to the area where their teammates were positioned. When bringing the ball back into play, almost all of the goalkeepers in this tournament were able to punt the ball deep into the attacking part of the pitch, but only a few goalkeepers were able to effectively reach their attackers.

This paper is a first step toward a more in-depth study of the technical and tactical actions of specific positions in women’s football in general and of goalkeepers in particular. It is necessary to mention the limitations of this study, due to the scarcity of studies that examine the role of the goalkeeper from a technical and tactical point of view (Di Salvo et al., 2008; García-Angulo and Ortega, 2015).

Information provided by the current research can enable a more thorough understanding of goalkeeper performance characteristics. Our consideration is in full compliance with Sainz de Baranda et al. (2005a) and Jara et al. (2018) who claim that the player should be trained to meet the requirements on the game. Currently, the goalkeeper progressively has a larger role in the collective game, so for a complete learning process, planning of goalkeeper practice should consider both attacking and defensive actions.

## Conclusion

This study allows us to identify, characterize and differentiate different attack and defense patterns of the goalkeeper between successful and unsuccessful teams. Results show that significant differences between the two groups. Data establishes that successful teams are characterized by an offensive game pattern with greater number of actions and more precision. On the other hand, goalkeepers on unsuccessful teams have shown a defensive game pattern with more defensive actions.

## Ethics Statement

This study respected the ethical principles established by the UNESCO Declaration on Bioethics and Human Rights. The study was approved by the Ethics Committee of University of Murcia (Spain) with ID 1944/2018.

## Author Contributions

All authors participated in the design, documentation, development, and writing of the manuscript. This paper was reviewed by all authors and all of them were responsible for its contents and providing they are responsible for the final version.

## Conflict of Interest Statement

The authors declare that the research was conducted in the absence of any commercial or financial relationships that could be construed as a potential conflict of interest.
